# POTENTIAL BENEFITS FROM LIVING IN A CCRC

**DOI:** 10.1093/geroni/igz038.816

**Published:** 2019-11-08

**Authors:** Anne Wyatt-Brown

**Affiliations:** University of Florida, Gainesville, Gainesville Florida, United States

## Abstract

Unlike many people who hope to postpone moving until late in life, my husband and I had to move quickly because he could no longer manage the stairs in our condo. We chose a nearby CCRC where my parents had lived in the 1980s. My husband loved our choice, and gradually I adjusted to life in an institution. When he died in 2012, I had to start over again as a single woman. Fortunately, people were very kind to me, which made adjusting much easier. Other residents were equally supportive when I had breast cancer, which involved chemo and radiation. I realized recently that co-residents have taught me to make supportive comments when people tell me that they can’t remember names. Acknowledging that we all have declines to deal with makes it easier for us all to accept aging’s inevitable changes while using our housing community for support.

